# Abrupt Increase in Detection of Locally Acquired West-Nile-Virus-Lineage-2-Mediated Neuroinvasive Disease in a Previously Non-Endemic Area of Southern Italy (2023)

**DOI:** 10.3390/v16010053

**Published:** 2023-12-28

**Authors:** Daniela Loconsole, Francesca Centrone, Anna Sallustio, Daniele Casulli, Vito Colella, Onofrio Mongelli, Giulietta Venturi, Antonino Bella, Leonardo Marino, Domenico Martinelli, Maria Chironna

**Affiliations:** 1 Hygiene Section, Department of Interdisciplinary Medicine, University of Bari, 70124 Bari, Italy; daniela.loconsole@uniba.it (D.L.);; 2 Hygiene Unit, Azienda Ospedaliero-Universitaria Consorziale Policlinico di Bari, 70124 Bari, Italy; 3 Department of Health Promotion and Animal Welfare, Apulia Region, 70124 Bari, Italy; 4 Department of Infectious Diseases, Istituto Superiore di Sanità, 00161 Rome, Italy; 5 Istituto Zooprofilattico Sperimentale della Puglia e della Basilicata, 71121 Foggia, Italy; 6 Hygiene Section, Medical and Surgical Sciences, University of Foggia, 71122 Foggia, Italy

**Keywords:** West Nile virus, West Nile neuroinvasive disease, Southern Italy, climate changes, one health, integrated surveillance

## Abstract

West Nile virus (WNV) is a public health concern in Europe. Rising temperatures and the migration of potential vectors promote the spread of viruses to previously unaffected areas. In 2023, the Apulia region of Southern Italy experienced an unexpected increase in West Nile neuroinvasive disease (WNND); no such cases had been reported in the previous 10 years. Overall, eight autochthonous cases of WNV infection were identified between July and October 2023, six of which were WNND. All cases were male (median age, 73 years). Two of the cases were blood donors. All WNND cases were hospitalized and all recovered within a few weeks. Surveillance data showed that, in the Apulia region, WNV Lineage 2 was detected in humans, mosquitoes, and horses. Based on the number of WNND cases reported, we can assume that a high number of infections occurred during the summer period. Changes in the climate in the region over recent years could be considered among the main drivers of the rapid increase in WNV infections. Therefore, integrated surveillance should be strengthened to avoid the potential massive spread of WNV in Southern Italy. Moreover, the implementation of whole-genome sequencing of WNV strains, as well as seroepidemiological studies in the area, will facilitate a better understanding of circulation dynamics.

## 1. Introduction

West Nile virus (WNV) is one of the most widespread viruses worldwide, representing a serious public health concern in many regions and countries. The transmission cycle involves wild birds and vector-competent mosquitoes, mainly belonging to the Culex (Cx.) genus [1]. Humans and horses represent incidental dead-end hosts [2]. Biological and socioenvironmental factors affect the dynamics of WNV spread, as well as the origin of epidemics [3]. Environmental factors have been considered as potential regulators of the annual incidence rate; in fact, increased temperatures shorten the replication cycle of vectors, and feeding increases accordingly (accompanied by increased biting frequency) [3]. Symptomatic disease is more common in horses than in humans, and the neuroinvasive form has a mortality rate of 33% [4]. Human cases are thought to be the tip of the iceberg since most are asymptomatic, and full recovery is usual [5,6]. With respect to symptomatic cases, 20% present with febrile illness (i.e., West Nile fever [WNF]), whereas less than 1% suffer neuroinvasive disease (West Nile virus neuroinvasive disease [WNND]); the latter is characterized by encephalitis, meningitis, and/or acute flaccid paralysis [2,7]. Neuroinvasive disease is more common in aged and immunocompromised patients [7]. More than 80% of patients with WNND require hospitalization, and almost two in three require admission to the intensive care unit [8,9]. Neurological complications carry a case fatality risk ranging from 3% to >20%, as well as life-long sequelae [6,7]. Viremia during WNV infections (and during infection by Flaviviruses) is of low level and short duration [3]. No vaccination or specific therapies are available for human cases; the treatment of WNV infection is supportive only [6].

At least nine lineages of WNV have been described worldwide, with Lineages 1 (L1) and 2 (L2) being the most prevalent in Europe [10]. WNV L1 was first introduced from Northern–Western African countries to Italy and France in 1988 and 2000, respectively, while WNV L2 was identified in Hungary in 2004 [4]; since 2008, it has spread across central Europe and the eastern Mediterranean region, becoming more virulent in vertebrates [11].

Currently, Europe has no specific surveillance programs to monitor seroprevalence, and testing is only indicated on clinical suspicion of infection [12]. However, the identification of WNV in animals (i.e., mosquitoes, horses, and birds) requires public health measures to prevent human-to-human transmission through contaminated blood donations [4]. According to the EU Blood Safety Directive, a negative individual donor nucleic acid test, or 28 days of deferral from donation, is required if the donor has returned from areas in which human cases of WNV infection have been detected [13]. From the beginning of the 2023 transmission season to 11 October 2023, EU/EEA countries reported 632 human cases of WNV infection, and 50 deaths [14]. In Italy, since 2008, an average of 60 human cases per year of WNV infection have been reported, with an increasing trend identified after the introduction of Lineage 2 in 2011 [15]. The majority of cases were identified during summer, mainly due to factors such as migratory birds and higher temperatures [16]. Most of the cases reported in recent years occurred in regions of Northern Italy (Veneto and Emilia Romagna), while only sporadic cases were reported in Southern Italy [17]. West Nile surveillance in Italy is regulated by the National Plan for Prevention Surveillance and Response to Arbovirus 2020–2025, which suggests that imported and autochthonous cases of WNV infections should be monitored throughout the year [18]. According to this system, three areas have been identified: (a) high-risk areas characterized by the endemic presence of Flaviviruses; (b) low-risk areas with the sporadic observation of Flaviviruses; and (c) minimum-risk areas where Flaviviruses have never been reported [18]. Overall, in Italy, 298 human cases of WNV infection have been reported since May 2023, and nearly 60% of these presented with neuroinvasive symptoms [17]. In the Apulia region (Southern Italy), the first two cases of WNND were identified in 2013. One patient reported that recent travel in the Italian Emilia Romagna region was the likely source of exposure (i.e., an autochthonous non-regional case); the other patient was classified as an autochthonous regional case. No other WNV infections, imported or autochthonous, have been reported in the Apulia region since 2013. Ten years later, linked to a Europe-wide spread of WNV infections, the local spread of WNV both in animals and humans was reported in the region.

The objective of the present study is to describe the unexpected occurrence and rapid increase of human cases of WNV neuroinvasive disease, and the spread of the virus in animal hosts, in the Apulia region of Southern Italy in 2023.

## 2. Materials and Methods

In the Apulia region, surveillance activities were performed jointly by the teams for infectious diseases at the Regional Department of Health, and at the Laboratory of Molecular Epidemiology and Public Health of the Hygiene Unit (A.O.U.C. Policlinico Bari), which is the regional reference center for WNV diagnosis. According to national guidelines, the Apulia region is classified as a low-risk area; therefore, surveillance comprises the following measures: controlling resident birds belonging to target species or rural or outdoor poultry herds; entomological surveillance; surveillance of nervous symptoms in Equidae; surveillance of specimens of wild birds found dead; and surveillance of cases of neuroinvasive disease and/or recent human infections [18]. Moreover, for all blood donors living in an area where the presence of WNV in animals was identified, a negative individual donor nucleic acid test for WNV identification is required. Cases were classified as probable or confirmed according to national guidelines [18]. For each suspected case of WNV infection, one sample of blood, one of serum, and one of urine, along with cerebrospinal fluid (CSF) in the case of WNND, should be collected, and a differential diagnosis to exclude Usutu virus infection is required. Confirmed WNV cases are defined as detection of viral RNA in blood, urine, and/or CSF. Detection of a specific antibody response in the absence of viral RNA requires confirmation by neutralization assay.

The data presented in the present study were collected from 1 January to 31 October 2023. Viral RNA was extracted from all available clinical samples using the Qiagen EZ1robot system (Qiagen; Milan, Italy) and tested using a commercial real-time PCR assay (Viasure West Nile Virus Real-Time PCR Detection Kit; CerTest Biotec S.L., Zaragoza, Spain). Negative samples were also tested for Usutu virus using a real-time PCR commercial kit (Quanty Usutu; Clonit, Milan, Italy). WNV Lineage 1 was discriminated from WNV Lineage 2 as previously described [19]. The presence of anti-WNV IgM and IgG antibodies in serum was detected using a commercial ELISA test (EUROIMMUN ITALIA s.r.l; Padova, Italy) and a commercial CLIA test (Virclia by Vircell; Granada, Spain). Samples of probable cases (positive for antibodies but negative for viral RNA) were further tested in the plaque reduction neutralization test (PRNT), which was performed at the Instituto Superiore di Sanità (Rome, Italy) as previously described [20]. Moreover, seroconversion was ascertained 14 days after collection of the first sample. Data on animal surveillance were retrieved from national reports on the integrated surveillance of West Nile and Usutu viruses [17], as updated by the Istituto Zooprofilattico Sperimentale di Puglia e Basilicata.

All activities conducted during the course of the study were part of the legislated mandate of the Health Promotion and Public Health Department of the Apulia region (Italy); therefore, approval by the Ethics Committee was waived. All procedures were performed in accordance with the Declaration of Helsinki, as revised in 2013, for research involving human subjects.

## 3. Results

**Figure 1:**
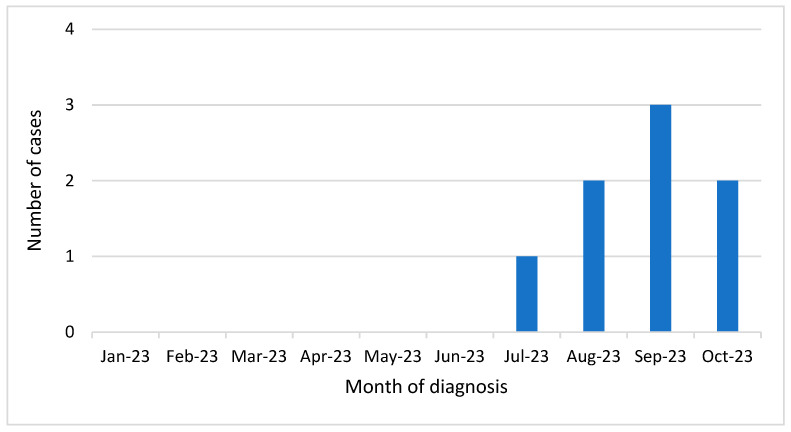
Distribution of WNV infections detected in the Apulia region (Southern Italy) from January–October 2023, presented according to month of diagnosis.

During surveillance from 1 January to 31 October 2023, eight cases of WNV infection, without an epidemiological linkage with each other, were identified in the Apulia region of Italy. All were identified between July and October (Figure 1).

**Figure 2:**
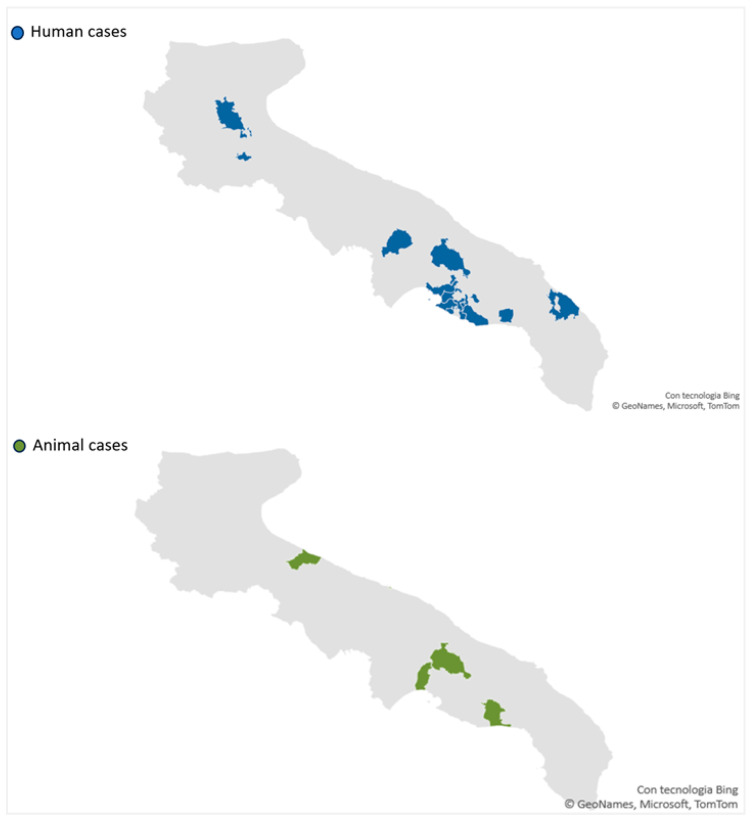
Geographic distribution of human cases and animal cases of WNV infection in the Apulia region (Southern Italy), January–October 2023.

All human cases were classified as autochthonous: six were WNND, and two were identified through the screening of blood donors (one WNF and one asymptomatic). The overall notification rate was 0.2 per 100,000 population. The median age of the cases was 73 years (IQR: 54.8–55.5), and all were male. None reported recent travel history, five patients were retired subjects, one patient was a police officer, while, for two patients, the occupation was not known. Moreover, three subjects lived in a rural area, and five in an urban area. Human cases were identified in four of six provinces in the region, while cases in animals were identified in the provinces of BAT (one pool of mosquitoes) and Taranto (two horses) (Figure 2). All WNND cases were hospitalized, but none required intensive care support. All recovered without long-term sequelae.

Table 1 shows the results of molecular and serological tests performed on available samples from the patients.

Two cases (patients 3 and 7) were negative for WNV RNA; therefore, PRNT tests were performed. Both neutralization assays were positive; therefore, both cases were classified as confirmed cases. The six samples that were positive for WNV RNA were tested to identify the lineage: all were WNV Lineage 2.

## 4. Discussion

Mosquito-borne diseases are an emerging threat in Europe. In recent years, global climate change, a dramatic expansion in the range of certain competent vectors, and the migration of people and goods have led to an increase in the transmission of viruses [21]. In particular, temperature and precipitation played a crucial role in WNV spreading in horses and humans [5]. In fact, due to climate changes, diseases transmitted by Aedes, Culex, and Anopheles mosquitoes will significantly impact human health, and, according to the World Health Organization, in the next decades, malnutrition, heat stress, and vector-borne diseases will cause an additional 250,000 deaths per year [22,23].

Since the beginning of the 2023 transmission season, Italy has experienced an increasing number of human WNV infections [14]. Even though the regions of Northern Italy were already considered endemic for WNV infections, in the Apulia region, in contrast, no other case of human infection was identified since the first autochthonous case reported in 2013. Ten years later, an abrupt increase in human WNV infections, particularly WNND cases, occurred between July and October 2023; therefore, the classification of the Apulia region was changed from low-risk to high-risk. The average temperature in the region during 2023 was higher than in previous years. A general increase in temperature over recent decades has resulted in changes to the climate in the Apulia region [24]. Accordingly, we can speculate that climate change likely promoted the spread of both competent vectors and WNV in the area, thus being considered among the main drivers of the rapid increase in WNV infections.

Following the identification of WNV circulation in the region, according to national guidelines, interventions aimed at reducing the risk of viral spread have been activated, such as targeted actions against the vector [18]. In particular, the activity of removing larval foci in the region has been intensified. In addition, communication was enhanced so that people living or working in the province involved in the outbreak were concerned and prone to take personal protection measures and combine forces to remove larval foci on their private property. After the implementation of these measures, no other cases of WNND have been identified throughout the region. Measures aimed at controlling vectors have been reported to play a more substantial role in reducing the prevalence of many mosquito-borne diseases compared with pharmaceutical measures or vaccines [25].

The neuroinvasive form of WNV infection occurs in less than 1% of cases [2,7]. In our region, six cases of WNND were identified in 2023, suggesting that hundreds of infections had occurred. Neuroinvasive disease is more evident in equines than in humans; therefore, equid cases could be used as indicators of viral activity to identify high-risk areas for humans [5]. Moreover, areas with cases identified only in horses may indicate an ongoing outbreak in humans that has not yet been detected [5].

The combined surveillance of mosquitoes, horses, birds, and humans would allow a more integrated and comprehensive approach to identifying WNV disease in endemic countries [26]. Based on data from the integrated surveillance in the Apulia region in 2023, we can speculate that a huge number of infections may have occurred, highlighting the need for stricter monitoring of WNV circulation in the coming years.

All cases described in the present study were male. A retrospective study conducted in Europe between 2006 and 2021 reported that more than 60% of cases were male [7]. Many studies reported a significant association between the male sex and a higher risk of developing WNND compared with asymptomatic disease [8]. Moreover, older age is a factor associated with WNND, which is in line with the median age of the cases identified in our study [7]. Moreover, 75% of our cases required hospital admission, a figure similar to that reported for the European region between 2006 and 2021 (71.4%) [7]. In the Apulia region, no deaths were reported during the epidemic of 2023. Currently, in Italy, 22 deaths have been reported, with a case fatality rate (CFR) of 6%, which is lower than the 22% reported during a large outbreak of WNV L2 that occurred in Northern Italy in 2018 [17,27]. In Europe, a CFR of 18.4% among hospitalized patients with WNND has been reported [7].

It is worth mentioning that two cases were identified through the screening of blood donors. WNV was the first arbovirus to emerge as a transfusion-risk pathogen [28]. However, a proactive approach to blood transfusion safety allows the identification of acutely infected, asymptomatic, and pre-symptomatic individuals, thereby ensuring blood transfusion safety [28]. The two cases detected herein confirm that such a screening is effective with respect to preventing post-transfusion infections.

Finally, our study revealed that all detected cases were WNV L2. The same lineage was identified in mosquitoes and animals [17]. This lineage was reported in Sub-Saharan Africa and Madagascar, before spreading to Europe, and no genetic flow in the opposite direction was reported [4]. Currently, in Italy, the circulation of both Lineage 1 and 2 has been documented [17]. Further analysis through the molecular characterization of WNV strains by whole-genome sequencing, coupled with seroepidemiological studies in the area, will allow for a better understanding of the phylogenetic relationships between Lineage 2 strains and circulation dynamics.

## 5. Conclusions

The rapid increase in the spread of arbovirus in a previously unaffected area such as Southern Italy is concerning. Climate change substantially affects the spread of vector-borne diseases. It is possible that the rising temperatures, along with the spread of mosquitoes and the movement of goods, could contribute to the possible introduction of new vectors and the rapid increase in WNV infections in Southern Italy. Strengthening the integrated surveillance system is crucial to ensure that we are prepared to face a potential massive spread of WNV in Southern Italy.

## Figures and Tables

**Table 1:** Characteristics of clinical samples obtained from patients infected with West Nile virus in the Apulia region (Southern Italy), January–October 2023.

		Molecular Test			Serological Test (ELISA and CLIA)		Seroconversion * (IgG)	PRNT	Lineage
		(Real-Time PCR)							
Patient	Classification of Case	CSF	Blood	Urine	IgM	IgG			
1	WNND	-	**+**	Na	**+**	**+**			2
2	WNND	**+**	-	**+**	**+**	-	**+**		2
3	WNND	-	Na	-	**+**	**+**		**+**	
4	WNF	Na	**+**	Na	-	-	**+**		2
5	WNND	-	Na	**+**	**+**	**+**			2
6	WNND	**+**	**+**	**+**	**+**	-	**+**		2
7	WNND	Na	-	-	**+**	-	**+**	**+**	
8	WNV infection	Na	**+**	-	-	**+**			2

WNND = West Nile neuroinvasive disease; WNF = West Nile fever; WNV = West Nile virus; CSF = cerebrospinal fluid; Na = not available; PRNT = plaque reduction neutralization test. * Sample collected 14 days after the first sample.

## Data Availability

The data are available upon request from the corresponding author.
